# Orthology Clusters from Gene Trees with *Possvm*

**DOI:** 10.1093/molbev/msab234

**Published:** 2021-08-05

**Authors:** Xavier Grau-Bové, Arnau Sebé-Pedrós

**Affiliations:** 1 Centre for Genomic Regulation (CRG), Barcelona Institute of Science and Technology (BIST), Barcelona, Catalonia, Spain; 2 Universitat Pompeu Fabra (UPF), Barcelona, Catalonia, Spain

**Keywords:** orthology inference, gene phylogenetics, comparative genomics

## Abstract

*Possvm* (Phylogenetic Ortholog Sorting with Species oVerlap and MCL [Markov clustering algorithm]) is a tool that automates the process of identifying clusters of orthologous genes from precomputed phylogenetic trees and classifying gene families. It identifies orthology relationships between genes using the species overlap algorithm to infer taxonomic information from the gene tree topology, and then uses the MCL to identify orthology clusters and provide annotated gene families. Our benchmarking shows that this approach, when provided with accurate phylogenies, is able to identify manually curated orthogroups with very high precision and recall. Overall, *Possvm* automates the routine process of gene tree inspection and annotation in a highly interpretable manner, and provides reusable outputs and phylogeny-aware gene annotations that can be used to inform comparative genomics and gene family evolution analyses.

## Introduction

Gene orthology inference is a central problem in genomics and comparative biology (Koonin 2005). Orthology information can serve as the basis for gene family classification, make inferences about gene function under the “ortholog conjecture” (Koonin 2005), enable cross-species comparisons, or trace the evolutionary dynamics of gene families, for example, looking for specific expansions or secondary losses (Glover et al. 2019). In addition to genome-scale methods (Altenhoff et al. 2016; Glover et al. 2019), a common orthology inference strategy involves the supervised construction of phylogenies, followed by manual curation in order to make informed inferences about gene family evolution. Yet, supervised tree inspection can be labor-intensive and difficult to scale.

Here, we present *Possvm* (Phylogenetic Ortholog Sorting with Species oVerlap and MCL [Markov clustering algorithm]), a flexible and accurate tool to identify pairs and clusters of orthologous genes (orthogroups) from precomputed phylogenies, and obtain inclusive gene family classifications. It relies on the species overlap algorithm (Huerta-Cepas et al. 2007, 2016) to identify pairs of orthologous genes in a phylogeny, and processes this output to identify groups of orthologs (defined as homologs descending from a single gene at the user-defined taxonomic scope), propagate gene name annotations, and report evolutionary relationships between gene pairs. *Possvm* can work with minimal input information: only a gene tree in *NEWICK* format, with or without node statistical supports. As the species overlap algorithm relies on the implicit taxonomic information contained in the gene tree’s topology, this approach is suitable for cases where the species tree is unknown or unavailable.

## New Approaches

**Fig. 1:**
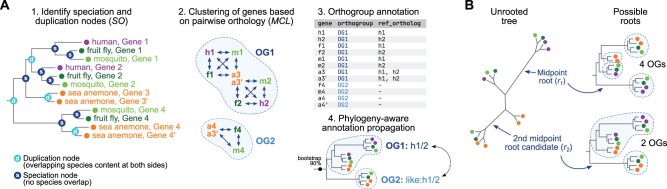
(*A*) Summary of the main steps in *Possvm*. The final step produces an annotated table with the orthology group assignments of each gene, as well as, optionally, their orthologs in a reference species (human in this example). (*B*) Example of the iterative midpoint rooting procedure. In this example, the original root (*r*_1_) results in the identification of four orthogroups whereas the second iteration (*r*_2_) results in two.

*Possvm* identifies pairs and clusters of orthologs (orthogroups) from a precomputed gene tree and propagates gene name annotations along the tree, in four main steps (fig. 1*A*). First, *Possvm* takes as input a gene tree where species are specified as a prefix to the gene name (e.g., *species id. | gene id.*), and identifies pairs of orthologous genes using the species overlap algorithm (Huerta-Cepas et al. 2007, 2016). By default, no overlap in species composition is tolerated at any bipartition (species overlap threshold = 0), but this parameter can be adjusted (where greater overlap will result in more inclusive, less granular orthology calls). Second, *Possvm* builds a graph where pairs of genes (nodes) are linked according to their orthology relationships (edges). If tree bipartitions contain statistical supports (e.g., bootstraps or Bayesian posterior probabilities), this graph can be pruned to remove poorly supported edges. This graph is partitioned into orthology clusters using MCL (Enright et al. 2002) and a user-defined inflation parameter (default is *I = *1.6) and, optionally, bootstrap supports as edge weights. This clustering strategy is commonly applied to binary protein networks such as protein–protein interaction graphs (Vlasblom and Wodak 2009). Thirdly, the software outputs a table with pairs of orthologous genes (from step one), a table with the orthogroup membership for each gene and its statistical support (step three), and an annotated gene tree with orthogroup labels next to each gene. Finally, *Possvm* can classify orthogroups using gene name information from one or more reference species, by propagating annotations across orthogroups in a phylogeny-aware manner (see below).

Optionally, the graph-based clustering step (fig. 1*A*) can be tweaked to focus on a subset of species. This option allows the user to build more accurate gene trees with well-sampled taxonomic outgroups (Zwickl and Hillis 2002), while restricting the orthology-calling step to the ingroup of interest. Following the example in figure 1*A*, the user could choose to include only bilaterian species in the analysis (human, mosquito, and fruit fly), which would result in the split of OG1 into two smaller, more granular orthogroups (as orthology edges leading to the sea anemone are ignored). This principle can be applied systematically to obtain hierarchical orthogroups and potentially improve precision (supplementary material S2, Supplementary Material online).

The species overlap algorithm requires a rooted gene tree in order to infer orthology relationships. Tree rooting can be done using a priori knowledge about taxonomic outgroups, using the midpoint rooting heuristics, or incorporating computationally intensive procedures such as nonreversible evolutionary models (Yap and Speed 2005; Bettisworth and Stamatakis 2021; Minh et al. 2020) and species tree reconciliation (Wu et al. 2014). *Possvm* offers the possibility to: 1) use precomputed rooted trees, 2) perform midpoint rooting, or 3) perform an iterative rooting procedure based on midpoint rooting, which selects a root based on an implicit parsimony criterion that minimizes the number of ancestral gene copies in a given tree. In this iterative rooting approach (fig. 1*B*), *Possvm* will start by identifying the midpoint root (initial root, *r*_1_) and call orthogroups from that topology; then, it will ignore the initial root node and try the second best midpoint root candidate (*r*_2_), up to *n* times (*r_n_*). Finally, it will select the root node that minimizes the number of orthogroups in the tree. The iterative rooting procedure can be suitable in cases where a long internal branch could be mistakenly selected as root by the standard midpoint approach.

In addition, *Possvm* can attempt to annotate genes and orthogroups using gene names from one or more reference species. For these steps, a dictionary file mapping the reference gene IDs (as used in the input gene tree) to the annotation of interest is required. First, individual genes are annotated as orthologous to one or more genes from the reference set. Second, the entire orthogroup is labeled according to the reference genes within, creating a composite name if it contains more than one reference gene (e.g., *name A/B*). Finally, orthogroups lacking any reference gene can be annotated according to their closest labeled orthogroups according to the tree topology (receiving a label in the following format *like: name A/B*). If available, *Possvm* will also report the statistical support for the deepest node in the phylogeny that supports a given annotation.

*Possvm* is freely available in Github (https://github.com/xgrau/possvm-orthology, last accessed August 10, 2021) under a GNU General Public License v3.0 license, together with test data and installation instructions. It requires Python 3 and the libraries *ETE*3 (Huerta-Cepas et al. 2016), *numpy* (Harris et al. 2020), *pandas* (McKinney 2010; Pandas Development Team 2021), *networkx* (Hagberg et al. 2008), and *markov_clustering* (Enright et al. 2002).

## Benchmarking the Accuracy of the Orthology Clustering

We used *Possvm* to classify orthologs in the ANTP homeobox class, a multigene family of transcription factors that is highly expanded in animals. This analysis allowed us to evaluate the accuracy of our orthology clusterings—using the manually curated ANTP families available in the HomeoDB database (Zhong and Holland 2011) as a reference—and probe the evolutionary history of ANTPs.

Our analysis included whole-genome sequences from 14 bilaterians (including reference species such as *Homo sapiens* and *Drosophila melanogaster*), 12 cnidarians, and two placozoans (supplementary material S1, Supplementary Material online). We used a standard pipeline commonly used in many gene family evolution studies: we used known ANTPs from the HomeoDB database to identify homologs in our genomes of interest using similarity searches (with *diamond*; Buchfink et al. 2015; 1,565 hits), built a multiple sequence alignment (*mafft* 7 *E-INS-i*; Katoh and Standley 2013), and a maximum-likelihood phylogenetic tree (*IQ-TREE* 2; Kalyaanamoorthy et al. 2017; Hoang et al. 2018; Minh et al. 2020).

**Fig. 2:**
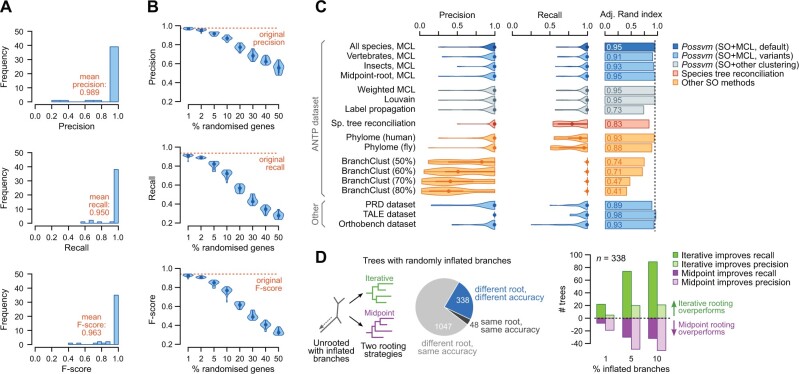
(*A*) Precision, recall, and *F*-score values for 43 ANTP families defined in HomeoDB. Mean values have been weighted by family size. (*B*) Effect of gene misplacement on precision, recall, and *F*-score, for the ANTP data set. (*C*) Distribution of accuracy statistics (precision, recall, adjusted Rand index) for the ANTP families, using various methods (details in supplementary material S2, Note 1, Supplementary Material online). (*D*) Effect of the iterative rooting strategy in precision, recall, and *F*-score, for the Orthobench tree collection. The pie plot shows the number of inflated pairs of trees that had the same or different roots and orthology solutions using each rooting strategy. The bar plots show how often did iterative or midpoint rooting improve recall or precision in the subset of trees with different roots and overall accuracies. Source data available in supplementary material S3, Supplementary Material online.

We evaluated the accuracy of *Possvm* against a curated classification of ANTP families in nine model bilaterian species available in HomeoDB (six vertebrates and three insects; see Methods in supplementary material S2, Supplementary Material online). *Possvm* identified a single orthogroup matching each of the 43 ANTP families in this reference set with high precision (weighted mean = 0.99) and recall (weighted mean = 0.95; fig. 2*A*; possible sources of error are discussed in supplementary material S2, Supplementary Material online). We also measured accuracy using all orthogroups containing a majority of genes from the reference families. This more inclusive metric results in higher recall without a detrimental effect on precision (supplementary material S3, Supplementary Material online). *Possvm* showed comparably high performance in other data sets, including subsets of insect and vertebrate ANTPs, the PRD and TALE homeobox classes, and 70 manually curated orthogroups from the Orthobench database (Trachana et al. 2011; Emms and Kelly 2020): in all cases, average precision and recall were above 0.90 (fig. 2*C* and supplementary materials S3 and S4, Supplementary Material online).

Given *Possvm*’s reliance on precomputed gene trees, its accuracy depends on the quality of the phylogenetic reconstruction. To evaluate how poorly constructed gene trees might affect *Possvm*’s orthology clusters, we randomized the position of tip nodes in the ANTP phylogeny (fig. 2*B*), finding that precision remained relatively high (above 0.75) even if we randomized up to 20% of node placements in the tree (i.e., 312 out of 1,565 genes). On the other hand, gene tree inaccuracies have a strong detrimental effect on recall (*ca.* 0.5 at 20%).

We have also used these data sets to compare *Possvm* with other gene tree-based orthogroup inference methods (fig. 2*C*, details in supplementary material S2, Supplementary Material online). Specifically, we have estimated precision, recall, and the adjusted Rand index, which reflects the similarity between clusterings (ours and the reference). The orthogroups that can be inferred the pairwise orthologies available in *PhylomeDB*, based on the species overlap algorithm but lacking a taxonomically unbiased clustering step (Huerta-Cepas et al. 2014), are similarly precise but have lower recall. A clustering step following a species tree reconciliation approach, which typically produces more fragmented pairwise orthology relationships (van der Heijden et al. 2007), resulted in lower recall as well. Finally, *BranchClust* (Poptsova and Gogarten 2007) exhibited a tendency to merge clusters and thus low precision (supplementary material S2, Supplementary Material online). Overall, combining species overlap with a clustering step resulted in the best combination of precision, recall, and clustering similarity to the reference families (fig. 2*C*).

Finally, we have also evaluated the effect of the iterative tree rooting strategy on orthology inference. This rooting heuristics often improved recall in a simulated set of gene trees with severe long-branch artifacts, albeit at the cost of occasional lower precision due to overclustering (fig. 2*D* and supplementary materials S2, S4, and S5, Supplementary Material online). Whether to undertake this approach or not thus depends on the intended goal of the analysis: when attempting to annotate as many homologs of a gene family as possible, it may be sensible to maximize recall at the risk of overclustering. In any case, the precision of pairwise orthology relationships within each orthogroup would be unaffected by the rooting strategy.

Together, these results indicate that *Possvm* faithfully approximates the manual process of tree inspection aimed at gene family classification, identifying a single orthology group that perfectly matches the reference annotations in most cases (e.g., 77% of the ANTP families, 67% of PRDs, and 85% of TALEs).

## Phylogeny-Informed Gene Annotation and Evolutionary Insights

**Fig. 3:**
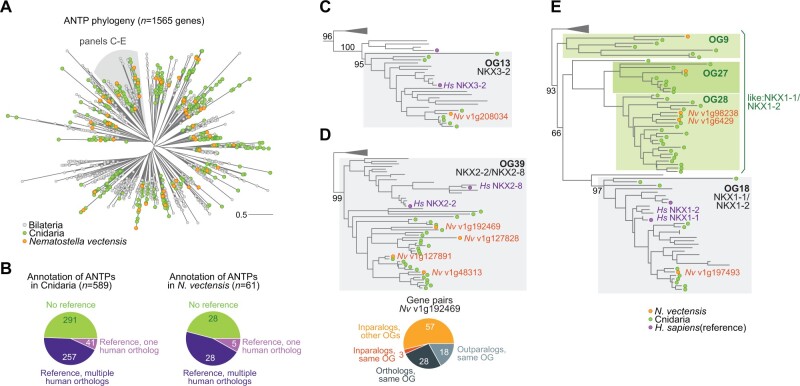
(*A*) Global phylogeny of ANTP genes in bilaterians, cnidarians, and placozoans. (*B*) Summary of annotated genes in Cnidaria and in *Nematostella vectensis*. (*C–E*) Three examples of *Possvm* annotations from the ANTP phylogeny, including reporting evolutionary relationships at the gene pair level.

*Possvm* is able to annotate orthogroups using gene names from a custom dictionary or a reference species. This functionality can be used to propagate gene annotations to nonmodel species in an orthology-aware manner, and inform the evolutionary history of a gene family. To illustrate this functionality, we annotated cnidarian ANTPs using human gene symbols as a reference (fig. 3). We find that *ca.* 50% of cnidarian ANTPs belong to orthogroups that can be labeled with one or more human genes within (fig. 3*A* and *B*). For example, out of 61 genes in the sea anemone *Nematostella vectensis*, *Possvm* annotates 33 genes as members of known ANTP families (fig. 3*B*). Among these, five are direct orthologs of a single reference gene (e.g., NKX3-2, fig. 3*C*), and 28 have one-to-many or many-to-many orthology relationships with human genes (e.g., *N. vectensis* encodes four co-orthologs of the human NKX2-2/NKX2-8 genes; fig. 3*D*).

A further 28 *N. vectensis* ANTPs belong to orthogroups that could not be assigned to any known ANTP class based on their contents. Yet, *Possvm* is still able to label them as close paralogs of other orthogroups that do contain known genes, by propagating the annotations of phylogenetically close known orthogroups. Oftentimes, these unannotated orthogroups genes reflect cnidarian-specific duplicates with a many-to-one relationships with known ANTP families. For example, the NKX1-1/NKX1-2 family, which contains both bilaterian and cnidarian homologs (fig. 3*E*), is closely related to three cnidaria-specific orthogroups that would be annotated as similar to NKX1-1/NKX1-2 by *Possvm* (labeled as *like: NKX1-1/NKX1-2*). The greediness of this annotation propagation procedure can be controlled by defining a minimum statistical support for the last common ancestor between the annotated and unannotated orthogroups.

Finally, *Possvm* can also report fine-grained evolutionary relationships at the gene level. For example, taking as a reference the *N. vectensis* gene *v1g192469* (NKX2-2/NKX2-8 family), *Possvm* classifies its homologs as orthologs, in-paralogs, or out-paralogs, within or without the same orthogroup (fig. 3*D*). By systematically reporting such relationships, we can dissect sets of homologous genes into precisely defined groups according to their evolutionary histories. This functionality allows the researcher to identify specific evolutionary patterns (e.g., intraorthogroup duplications in a specific species), or to address evolutionary hypotheses in cross-species comparisons (e.g., testing the functional conservation of orthologous gene pairs compared with closely related paralogs).

## Discussion

*Possvm* is an accurate tool to automate the process of phylogeny parsing, ortholog clustering, and gene name annotation propagation, requiring a gene tree as its sole input. Importantly, the species overlap algorithm (Huerta-Cepas et al. 2007, 2016) that sits at its core emulates a common heuristics used by researchers when inspecting a gene tree: it is assumed that a certain degree of taxonomic coherence should be present within an orthology group, but that small-scale inaccuracies in the tree inference might introduce discrepancies with the underlying species phylogeny. Therefore, these orthology classifications are highly interpretable when visualized over the gene tree—that can be produced by *Possvm*, together with table-based annotations—, which facilitates its critical appraisal by the researcher.

We have demonstrated that *Possvm* classifications show very high precision and recall against a notably large multigene family (ANTP homeoboxes) and a curated benchmark of orthology groups (Trachana et al. 2011; Emms and Kelly 2020). Yet, it is crucial to highlight that *Possvm*’s performance depends on the quality of the input gene tree. In that regard, we have demonstrated that by combining the species overlap algorithm with MCL we can tolerate relatively high rates of gene misplacement in the phylogenies and still maintain reasonable precision (fig. 2*C*), and that the iterative rooting procedure can alleviate recall issues related to the presence of internal long branches in the gene tree (fig. 2*D*).

In recent years, we have witnessed a rapid increase in the taxonomic sampling and quality of whole-genome sequencing efforts. Similarly, functional genomics data such as single-cell transcriptomic atlases are now available for diverse species (Tanay and Sebé-Pedrós 2021). In that regard, *Possvm* provides an accurate and interpretable gene orthology inference solution that will facilitate gene family evolution studies, cross-species data integration, and large-scale comparative and functional genomics analyses.

## Supplementary Material

Supplementary data are available at *Molecular Biology and Evolution* online.

## Supplementary Material

msab234_Supplementary_Data
