# Supplementary material for: Orthology Clusters from Gene Trees with Possvm
Source: Mol Biol Evol. 2021 Aug 5;38(11):5204–8. doi: 10.1093/molbev/msab234 (PMC8557443; doi:10.1093/molbev/msab234)
Supplement: msab234_Supplementary_Data [file msab234_supplementary_data.zip › SM_S2_supplementary_notes.pdf]

Supplementary Notes

Supplementary Material S2

Grau-Bové and Sebé-Pedrós, 2021

Table of contents

Note 1. Methods 1

Note 2. Discussion of *Possvm* performance 6

Note 1. Methods

1.1. Data retrieval and gene tree inference

We have estimated the performance of *Possvm* and other orthology inference methods relative to two manually curated datasets: Orthobench (v2) (Trachana et al. 2011; Emms and Kelly 2020), and the ANTP, PRD and TALE classes of homeobox transcription factors from the HomeoDB database (Zhong and Holland 2011).

Orthobench consists of 70 reference orthogroups defined from manual examination of gene trees build from sequences of 12 complete bilaterian genomes. In this case, we retrieved the 'raw' gene trees of each of the 70 reference orthogroups (RefOGs) available in the database (https://github.com/davidemms/Open_Orthobench/). We used the provided *RefOGs.csv* orthogroup annotation file as a reference for the classification performance analyses (see below).

The HomeoDB database (http://homeodb.zoo.ox.ac.uk/) contains curated annotations of homeobox transcription factors in bilaterians (including five vertebrates, the amphioxus *Branchiostoma floridae*, and three insects). We used the family-level annotations of the three largest homeobox classes in the database

(ANTP, PRD and TALE) to define reference orthogroups.

In the case of the HomeoDB datasets, no reference gene tree is provided. Instead, we built our own trees using an unsupervised standard approach, commonly used in many gene family evolution studies. First, we downloaded all genes of the ANTP, PRD and TALE classes, and used them as seed sequences in separate homology searches against the predicted proteomes of 28 animal genomes (using the *diamond blastp* local aligner v0.9 (Buchfink et al. 2014), with the *--more-sensitive* search parameter). The resulting homology hits (filtered at *e*-value < 10^{-6}; 1,565 genes for ANTPs, 880 for PRDs, 246 for TALEs) were aligned with *mafft E-INS-i* v7 (Katoh and Standley 2013). We trimmed this alignment with *ClipKIT* (retaining parsimony-informative and constant sites and removing sites with a gap threshold over 0.7) (Steenwyk et al. 2020), and built a maximum-likelihood phylogenetic tree with *IQ-TREE 2* (Kalyaanamoorthy et al. 2017; Hoang et al. 2018; Minh et al. 2020) (up to 10,000 refinement iterations until 0.99 convergence threshold is achieved; UFBS statistical supports from 1,000 replicates; substitution model selected by ModelTest according to the Bayesian Information Criterion, resulting in JTT+Γ4 for ANTP and PRD, and JTT+Γ4+F+I for TALE).

1.2. Benchmarking of orthology inference methods

We have assessed the performance of various orthogroup inference methods based on gene tree analysis: (i) *Possvm*, (ii) *BranchClust* v1 (Poptsova and Gogarten 2007), (iii) a naive approach based on defining orthology groups from pairwise orthology relationships with a reference species (i.e., without a clustering step), in the manner of the orthology inferences available in the *PhylomeDB* database (Huerta-Cepas et al. 2007; Huerta-Cepas et al. 2014); and (iv) a strict species tree reconciliation procedure.

All methods have been tested using the same input gene trees (either retrieved from Orthobench, or our own maximum-likelihood trees of ANTP, PRD and TALE homeoboxes).

1.2.1. *Possvm*

Possvm has been tested on the ANTP, PRD, TALE and Orthobench datasets using a range of approaches: the iterative rooting procedure (*i* = 10 rooting iterations), standard mid-point rooting, and alternative strategies for the post-species overlap clustering step (statistical supports-weighted MCL, Louvain

clustering, and the label propagation algorithm; implementations from the Python *networkx* library, *community* submodule (Hagberg et al. 2008)).

For the tree of ANTP genes (HomeoDB), we assessed *Possvm*'s accuracy in three additional setups. Firstly, we used two different taxonomically-defined subsets of reference species from HomeoDB: five vertebrates (*Danio rerio*, *Gallus gallus*, *Homo sapiens*, *Mus musculus* and *Xenopus tropicalis*), and three insects (*Drosophila melanogaster*, *Apis mellifera*, *Tribolium castaneum*). Secondly, we also analysed the effect of inaccuracies in gene tree reconstruction, using a collection of randomised gene trees based on the original ANTP phylogeny. Specifically, we selected a fraction of genes pairs in the tree and permuted their placements (1%, 2%, 5%, 10%, 20%, 30%, 40%, and 50% of the genes in the original trees, using 20 replicates for each set).

For the Orthobench gene trees, we also assessed the effect of the iterative root selection procedure on orthology inference. This analysis requires creating a collection of orthology-curated gene trees with branch-length issues that could potentially be ameliorated with each rooting strategy. Unfortunately, this is a rare scenario in the Orthobench dataset (only 8 out of 70 trees had different roots and orthology solutions depending on the rooting strategy), which prevents a proper quantitative assessment. To obtain such a dataset, we used the Orthobench tree collection to build a simulated set of trees with severe long-branch artifacts by inflating the length of randomly selected branches in the input trees. Specifically, we build 20 randomly inflated trees per input tree, by lengthening 1% of their branches to the 99th quantile of the global branch length distribution. Then, we assessed the precision, recall and *F*-score of *Possvm* on the final, non-redundant tree collection, using mid-point and iterative rooting ($i = 10$). This procedure was repeated using 5% and 10% of the branches.

All tree manipulations (branch permutation and branch inflation) were handled in *R*, using the *ape* 5.4 (Paradis and Schliep 2019) and *phangorn* 2.5.5 packages (Schliep 2011).

1.2.2. *BranchClust*

The *BranchClust* algorithm requires that the user defines a minimum number of species per orthology group. Per the authors' recommendation (Poptsova and Gogarten 2007), we assessed four possible values

for this parameter (50%, 60%, 70% and 80% of the total number of species in each dataset). This parameter is critical to define the inclusiveness of *BranchClust*'s clusters: higher minimum number of species will typically result in more aggregative clusters (Figure 2C and Supplementary Material S4C) and the optimal value will depend on how prone a particular gene family is to secondary losses within an orthogroup. As it must be set once for each tree, this can result in suboptimal clusterings in gene trees where certain orthogroups are more prone to secondary losses than others.

1.2.3. *Naive orthology groups from species overlap (PhylomeDB-style)*

PhylomeDB-style orthology groups were obtained using human and fruit fly as reference species. This analysis was based on the *ETE3* toolkit (Huerta-Cepas et al. 2016) implementation of the species overlap algorithm (same as *Possvm*). In this case, orthogroups were defined as encompassing all genes that were orthologous to the genes from the focal species.

1.2.4. *Species tree reconciliation*

We used the species tree reconciliation function from the *ETE3* toolkit (Huerta-Cepas et al. 2016) to obtain a graph of pairwise orthology relationships between genes, and built orthology groups with an *MCL* clustering step (same as *Possvm*).

1.3. Classification performance metrics

We measured the performance of each orthology assignment method in terms of its precision (positive predictive value), recall (true positive rate), *F*-score (harmonic mean of precision and recall), and the adjusted Rand index (a measure of similarity between two clustering solutions that does not require equivalent labels in the query and reference sets). These scores were estimated for each reference orthology group by counting true positives (genes from a reference orthogroup that were correctly assigned to the same *Possvm*-derived cluster), false positives (genes from a different reference orthogroup assigned to that *Possvm*-derived cluster), and false negatives (genes from the reference orthogroup assigned to a different *Possvm*-derived cluster). The average value of each score in each dataset (ANTP, PRD, TALE, and Orthobench) is given as a weighted mean normalised by reference orthogroup size.

In order to decide which *Possvm*-derived cluster corresponded to each of the reference orthogroups, we

used two comparison strategies: (i) a 'best hit' approach that took into account the largest *Possvm*-derived cluster whose contents overlapped with the reference; and (ii) an inclusive approach that took into account all *Possvm*-derived clusters where a majority of genes overlapped with the contents of the reference group (Supplementary Material S4A).

These statistics were calculated in base *R*. The adjusted Rand index was calculated using the corresponding function in the *R fossil* 0.4.0 library (Vavrek 2011).

1.4. Data and code availability

All the data (gene trees, orthology assignments, etc.) and scripts required to reproduce our accuracy benchmark analyses are available in the following GitHub repository, together with step-by-step instructions: https://github.com/xgrau/possvm-orthology-benchmarking.

Note 2. Discussion of *Possvm* performance

In this section, we discuss two possible source of error in *Possvm* solutions (over-splitting of gene families resulting in low recall and over-aggregation resulting in low precision) with relevant examples from the ANTP gene family, as well as the effect of the rooting strategy in the Orthobench dataset.

2.1. Low recall due to orthogroup over-splitting

Out of 43 ANTP families, *Possvm* identifies 39 of them with maximum recall (i.e., all members in the reference dataset are included in the same *Possvm* group). There are six families where some members are missing: *Hox3*, *Hox5*, *Hox6-8*, *Nk3*, *Nk4*, and *Noto*. In all cases, low recall is due to the over-splitting of the reference group into smaller groups.

The root causes of over-splitting could be inaccuracies in the gene tree, or the existence of additional diversity within these gene families that was unaccounted for in previous classifications, as they relied on less extensive taxon samplings (note that our analysis includes a rich sampling of cnidarian and placozoan outgroups). For example, the *Noto* family appears split in three OGs (OG57, OG56 and OG41; recall = 0.41 relative to the largest group, OG57). This over-splitting could be caused by the actual paraphyly of the *Noto* family in bilaterians, given that there are multiple cnidarian- and placozoan-specific *Noto*-like clades branching among the reference *Noto* genes annotated in the HomeoDB database (Supplementary Material S5A). This could also be the case for *Nk3*, split into two OGs with a sister-group relationship (OG12 and OG13), one of which (OG13) has cnidarian members (Supplementary Material S6).

2.2. Low precision due to orthogroup merging

On the other hand, there are ANTP four families with precision less than 100%, due to their inaccurate merging in two orthogroups: OG0, which includes *Dbx* and *Hlx* genes; and OG11, which includes *Nanog* and *Ventx* (Supplementary Material S3). In both cases, this is due to the existence of intra-bilaterian paralogous clades that are merged in a single orthogroup due to their sister-group relationships with well-sampled clades of cnidarian genes.

For example, the OG0 orthogroup (including *Dbx* and *Hlx*) includes a well-sampled clade of cnidarian

genes that has a sister-group relationship with two mostly-bilaterian clades, corresponding to *Dbx* and *Hlx* (Supplementary Material S5B). This results in a dense orthology graph spanning cnidarian and the two bilaterian clades, which is interpreted by *Possvm* as a single orthology group (i.e., a gene family present as one copy in the cnidarian + bilaterian ancestor). In such cases, it is possible to recapitulate distinct orthogroups for the *Dbx* and *Hlx* families the bilaterian root by excluding cnidarian sequences from the orthology graph (*Possvm* parameter: *--outgroup <list of cnidarian species>*; see Supplementary Material S5B).

2.3. Improving accuracy: curation of the orthology graph and clustering strategies

Possvm offers various options to curate the graph of orthologous gene pairs obtained from the species overlap step. First, the species overlap algorithm offers a natural way to increase the inclusiveness of the final orthology graph by increasing the species overlap threshold parameter (i.e., the fraction of overlapping species at both sides of a tree bipartition that trigger its classification as a duplication event, *--sos <NUM>* flag; default is 0). Second, it is possible to remove all sequences from one or various species (using the *--outgroup* flag), which can be used to define a set of outgroups of blacklisted species. Third, if the gene tree contains node bipartition statistical supports, these can be used to ignore specific edges in the orthology graphs by filtering out pairs of orthologs with poorly supported shared ancestral nodes (by setting a support threshold with the *--min_support_node <NUM>* flag).

In practice, these options can be used to improve the accuracy of the final orthology groups, but their convenience will depend on the specific tree at hand and should be chosen upon careful examination of the resulting annotation. Supplementary Material S5B provides an example of how filtering out certain species (in this case, taxonomical outgroups) from the orthology graph can increase precision. Such approaches could also be used to improve recall if one or more fast-evolving species or sequences were found to incorrectly branch within an otherwise correct orthogroup, resulting in its splitting.

Finally, it is also possible to use phylogenetic statistical supports as edge weights in the MCL clustering step (*--method mclw* flag), so as to decrease the weight of certain pairwise orthologies (rather than/in addition to ignoring them altogether). In combination with increased *MCL* inflation values (*--inflation <NUM>* flag), this option can be used to obtain more granular, and therefore potentially precise, orthology clusters. By default, *Possvm* clusters binary orthology graphs (i.e. unweighted) with *MCL*, which tends to

produce more inclusive groupings, as all edges are included irrespectively of their statistical support. Given that these orthology graphs tend to be highly modular (with a few clusters having edges to/from other orthology groups), other modularity-based clustering strategies, such as Louvain, also perform well in this regard (Figure 2C, Supplementary Material S4B).

2.4. Effect of the rooting strategy

We have also assessed the effect of our iterative tree rooting strategy on *Possvm*'s accuracy. Given that the iterative rooting procedure selects the root that minimises the total number of orthogroups in the tree, this strategy could potentially increase recall at the cost of reduced precision, due to its tendency to produce more aggregative clusterings.

We compared the accuracy of *Possvm* in both sets of trees where the two strategies identified different roots (relative to the single best *Possvm*-derived orthogroup in each one), and found that orthology solutions from iteratively rooted trees exhibited higher recall in 5 out of 17 trees (Figure 2D and Supplementary Material S4C, D), reaching >80% recall in all five cases (Supplementary Material S3). In three additional trees, iterative rooting resulted in a reduction in precision that was not offset by the improvement in recall (difference in *F*-score iterative – midpoint < 0). These qualitative results suggest that the tree rooting strategy presents a trade-off between precision and recall.

In order to quantify this trade-off, we require a large set of gene trees with skewed long-branch distributions that might confound the rooting strategy (e.g. trees with very long internal branches). This requirement appears to be relatively rare in the Orthobench dataset, which consists of small, curated trees. To overcome this limitation, we generated a randomised collection of trees with severe long-branch issues based on the original Orthobench dataset (see Methods and Supplementary Material S4C, D), and measured the classification accuracy with both strategies. Most trees exhibited no change in the accuracy of their orthology classifications. However, amongst trees where each method selected different roots, iterative rooting resulted in higher recall more than three times as often (185 trees) than midpoint rooting (70 trees). Conversely, iterative rooting resulted in precision improvements in fewer cases (46 trees) than midpoint rooting (119 trees). Overall, these results suggest that iterative rooting is an efficient strategy to maximise recall, which might be a good strategy if the researcher aims to obtain aggregative gene family

classifications to annotate as many genes as possible.

The ANTP phylogenetic analysis provides a clear example of the effect of the tree rooting strategy on the species overlap algorithm (Supplementary Material S5A): based on the classical mid-point rooting strategy, *Possvm* infers 97 different orthogroups from the tree; whereas the iterative rooting procedure brings down this number to 94 at the second iteration. This difference is due to the splitting of the *Noto* homeobox family into three paraphyletic clades in the mid-point root strategy, caused by a very long branch in just one gene (*Saccoglossus kowalevskii* gene Skow_NM_001171216.1; Supplementary Material S5A). In this mid-point rooted tree, other bilaterian NOTO homologs (e.g. the *Drosophila melanogaster* FBtr0083571 gene) are further split into a fourth paraphyletic clade. On the other hand, in the iteratively rooted tree, the human, mouse and *S. kowalevskii* NOTO homologs form a monophyletic clade with various cnidarian genes (UFBS support = 79%), which has a sister-group relationship (UFBS = 80%) with the other known bilaterian NOTO genes (*D. melanogaster* FBtr0083571 gene).

Given that this discrepancy only affects species that are not part of the ANTP reference database (*S. kowalevskii* and various cnidarians), it does not have an effect on the overall accuracy. Yet, it highlights how the pitfalls of mid-point rooting could (i) result in the paraphyly of the various orthology groups that are part of the well-established *Noto* ANTP family (Zhong and Holland 2011); and (ii) result in a failure to detect *Noto* orthologs in specific species (in this case, *S. kowalevskii* and various cnidarians).

References

Buchfink B, Xie C, Huson DH. 2014. Fast and sensitive protein alignment using DIAMOND. *Nat Methods* 12:59–60.

Emms DM, Kelly S. 2020. Benchmarking Orthogroup Inference Accuracy: Revisiting Orthobench. *Genome Biol Evol* 12:2258–2266.

Hagberg AA, Schult DA, Swart PJ. 2008. Exploring Network Structure, Dynamics, and Function using NetworkX. In: Varoquaux G, Vaught T, Millman J, editors. Proceedings of the 7th Python in Science Conference. Pasadena, CA USA. p. 11–15.

Hoang DT, Chernomor O, von Haeseler A, Minh BQ, Vinh LS. 2018. UFBoot2: Improving the Ultrafast Bootstrap Approximation. *Mol Biol Evol* 35:518–522.

Huerta-Cepas J, Capella-Gutiérrez S, Pryszcz LP, Marcet-Houben M, Gabaldón T. 2014. PhylomeDB v4: Zooming into the plurality of evolutionary histories of a genome. *Nucleic Acids Res* 42:897–902.

Huerta-Cepas J, Dopazo H, Dopazo J, Gabaldón T. 2007. The human phylome. *Genome Biol* 8:R109.

Huerta-Cepas J, Serra F, Bork P. 2016. ETE 3: Reconstruction, Analysis, and Visualization of Phylogenomic Data. *Mol Biol Evol* 33:1635–1638.

Kalyaanamoorthy S, Minh BQ, Wong TKF, Von Haeseler A, Jermiin LS. 2017. ModelFinder: Fast model selection for accurate phylogenetic estimates. *Nat Methods* 14:587–589.

Katoh K, Standley DM. 2013. MAFFT multiple sequence alignment software version 7: improvements in performance and usability. *Mol Biol Evol* 30:772–780.

Minh BQ, Schmidt HA, Chernomor O, Schrempf D, Woodhams MD, von Haeseler A, Lanfear R. 2020. IQ-TREE 2: New Models and Efficient Methods for Phylogenetic Inference in the Genomic Era.Teeling E, editor. *Mol Biol Evol* 37:1530–1534.

Paradis E, Schliep K. 2019. ape 5.0: an environment for modern phylogenetics and evolutionary analyses in R.Schwartz R, editor. *Bioinformatics* 35:526–528.

Poptsova MS, Gogarten JP. 2007. BranchClust: A phylogenetic algorithm for selecting gene families. *BMC Bioinformatics* 8:1–16.

Schliep KP. 2011. phangorn: Phylogenetic analysis in R. *Bioinformatics* 27:592–593.

Steenwyk JL, Buida TJ, Li Y, Shen XX, Rokas A. 2020. ClipKIT: A multiple sequence alignment trimming software for accurate phylogenomic inference. *PLoS Biol* 18:1–17.

Trachana K, Larsson TA, Powell S, Chen WH, Doerks T, Muller J, Bork P. 2011. Orthology prediction methods: A quality assessment using curated protein families. *BioEssays* 33:769–780.

Vavrek MJ. 2011. fossil: palaeoecological and palaeogeographical analysis tools. *Palaeontol Electron* 14:1T.

Zhong Y, Holland PWH. 2011. HomeoDB2: functional expansion of a comparative homeobox gene database for evolutionary developmental biology. *Evol Dev* 13:567–568.
