# Supplementary material for: Orthology Clusters from Gene Trees with Possvm
Source: Mol Biol Evol. 2021 Aug 5;38(11):5204–8. doi: 10.1093/molbev/msab234 (PMC8557443; doi:10.1093/molbev/msab234)

Supplementary Material S5

A)

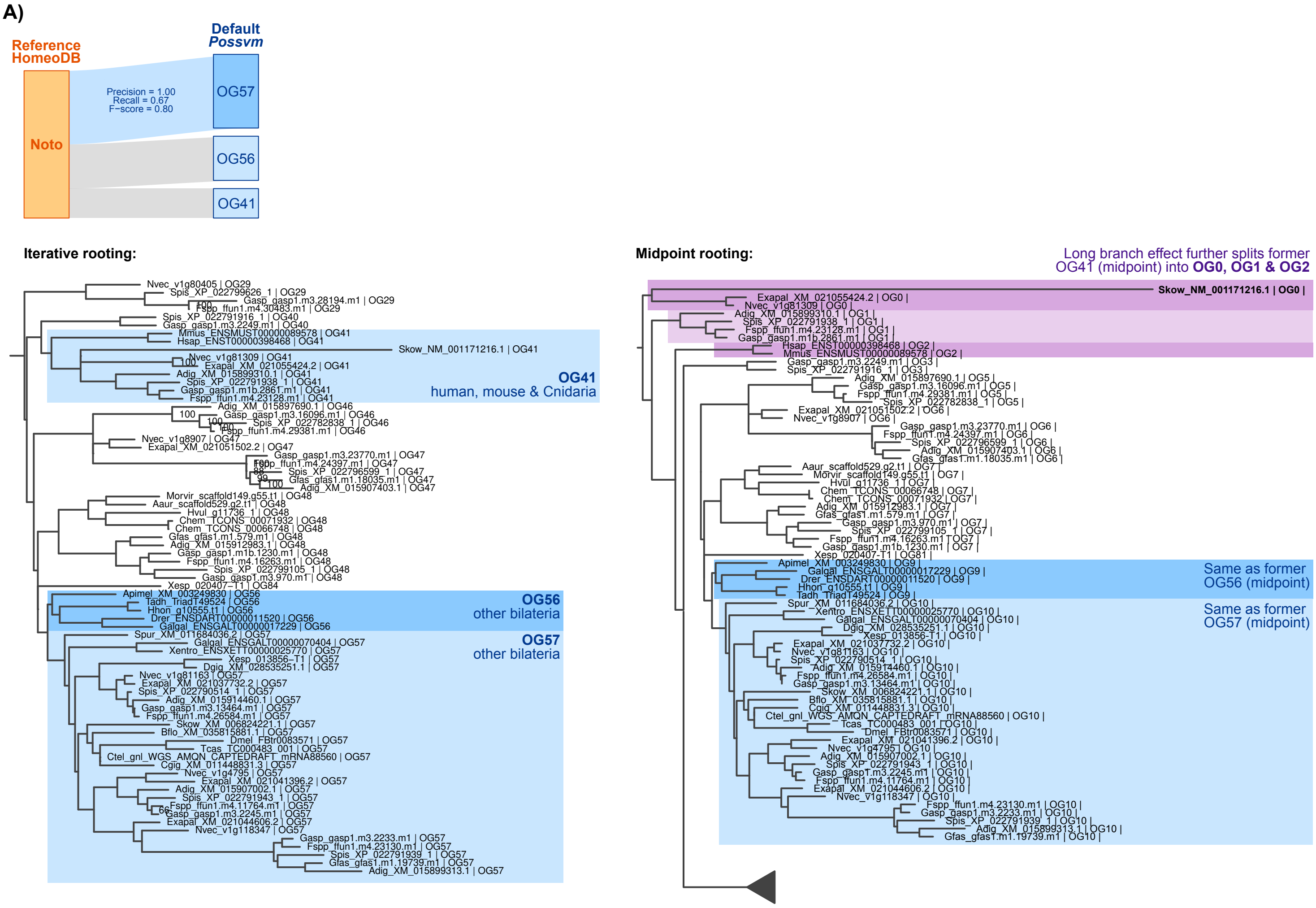

B)

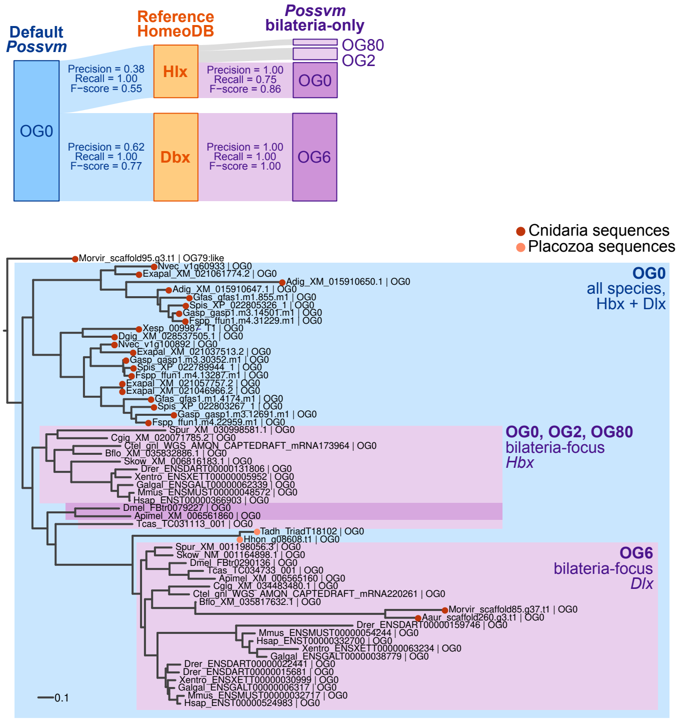

Supplement: msab234_Supplementary_Data [file msab234_supplementary_data.zip › SM_S5_diagnostics.pdf]
